# Dangers of Sulphonal

**Published:** 1890-03-29

**Authors:** 


### THERAPEUTICS.

### DANGERS OF SULPHONAL.

We gave, some weeks since, the experience of several eminent French, German, and Italian physicians as to the dangers attending the use of this new hypnotic, and showed from experiments on animals that its primary action was a toxic one on the spinal cord, considerations which, taken in conjunction with the late manifestation of its hypnotic effects, should render one extremely cautious in its employment, notwithstanding the sanguine expressions of opinion on the part of other observers. Dr. Umpfenbach, Resident Medical Officer of the Rhineland Lunatic Asylum at Andernach, must now be added to the number of those who look on it with distrust. He finds that sulphonal in doses of 1—2 grams daily induces a series of unpleasant, if not positively dangerous symptoms, such as palpitation and weakness of the heart's action, coldness and lividity of the extremities, tremors, partial paralysis, unsteadiness in walking, ataxy of the arms and hands, difficulty in articulation and disturbed intellect. Chloralamid he finds almost as unsatisfactory, and both far inferior to chloral hydrate.

